# Cue-based feeding in the NICU—a pathway to earlier oral feeding of preterm infants

**DOI:** 10.3389/fped.2024.1417628

**Published:** 2024-09-20

**Authors:** Noa Ofek Shlomai, Chen Mordechai, Iris Morag, Tali Bdolach Abram, Smadar Eventov Friedman

**Affiliations:** ^1^ Department of Neonatology, Hadassah Medical Center, Faculty of Medicine, Hebrew University of Jerusalem, Jerusalem, Israel; ^2^ Faculty of Medicine, Hebrew University of Jerusalem, Jerusalem, Israel; ^3^ Department of Military Medicine and “Tzameret”, Faculty of Medicine, Hebrew University of Jerusalem and Medical Corps, Israel Defense Forces, Jerusalem, Israel; ^4^ Department of Neonatology, Shamir (Assaf Harofeh), Zeriffin, Israel; ^5^ Department of Pediatrics, Sackler Faculty of Medicine, Tel Aviv University, Zriffin, Israel

**Keywords:** neonatal intensive care unit (NICU), cue-based feeding, volume-driven feeds, weight gain, oral feeds

## Abstract

**Aim:**

To compare volume-driven and cue-based feeding of low birth weight preterm infants, regarding short-term outcomes, including transition to oral feeds, weight gain, and length of stay.

**Methods:**

This was a retrospective cohort study. Feeding and weight gain outcomes were compared between infants fed by volume-driven and cue-based feeds. The groups were subdivided by birth weight categories.

**Results:**

The study group included 240 low birth weight preterm infants born before 34 weeks of gestation, 120 infants fed by volume-driven feeding were compared to 120 infants fed by cue-based feeding. The groups were sub-analyzed by birth weight categories: <1,500 g and 1,500–2,500 g. Study groups were comparable regarding baseline characteristics and neonatal morbidities. Infants fed by cue-based feeding were more likely to achieve full oral feeding faster and at an earlier gestational age. Infants with a birth weight <1,500 g were less likely to experience adverse respiratory episodes during cue-based feeding. Although the rate of weight gain was reduced in cue-based feeding in the heavier infant group, discharge weight, breastfeeding rates, and length of stay were comparable between the groups.

**Conclusions:**

Cue-based feeding results in faster transition to full oral feeding in very low birth weight preterm infants and at an earlier gestational age.

## Key point

• Transition to oral feeding is a challenge for low-birth-weight preterm infants.
• Cue-based feeding is a feeding method which is responsive to the infants' cues of satiety and hunger.
• Cue-based feeding results in faster transition of low-birth-weight infants to full oral feeding, at an earlier gestational age, with increased cardiorespiratory stability.

## Introduction

Prematurity, defined as birth before completing 37 weeks of gestation, compromises approximately 10% of live births worldwide (1). Discharge of preterm infants from the neonatal intensive care unit (NICU) depends on achieving certain milestones, such as respiratory stability, thermoregulation, oral feeds, and steady weight gain. In many cases, satisfied feeding pattern and desired oxygenation reserving are frequently achieved last and thus delay the discharge of preterm infants (2). Multiple studies demonstrated an association between prolonged hospital stay and delays in both physical and neurological development, as well as abnormal parent-infant bonding and interaction, and increased healthcare costs (3, 4).

Oral feed readiness requires coordination between suck, swallow, and respiration and usually occurs at 34–36 weeks of gestational age (GA) (2, 5). Over the years, many interventions aimed at facilitating the transition from nasogastric tube to oral feeding in preterm infants have been attempted. These include massage therapy, visual stimulation (6), music therapy (7), non-nutritive sucking (8), oral sensory-motor stimulation (9), self-paced feeding (10), and cue-based, or responsive, feeding (11). Importantly, preterm infants with prolonged naso/oro-gastric tube feeding are at an increased risk for later oral aversion (12).

Traditional feeding in the NICU includes a preset volume administered in scheduled intervals. The preset volume is calculated based on the infant's GA, weight, postnatal age, and other neonatal physiological parameters and morbidities. Traditional feeding, i.e., volume-driven feeds, discards the infant's sleep/awake state and hunger/satiety cues (11, 12).

On the other hand, cue-based feeding is an important component of the developmental care approach, which became the standard of care in NICUs. Developmental care in the NICU aims to improve the short- and long-term outcomes of preterm infants. This approach leans on acknowledging the infant's behavioral cues and using them for formulating an individual care plan rather than planning ahead in a “one-size-fits-all” fashion (12). In terms of feeding, the developmental care approach allows the infants a span of volume administered every 2–4 h, in response to the infant's cues. If the infant has not reached a minimal predetermined volume, a supplementation is given via nasogastric tube (11). Cue-based feeding is designed to reduce the stress and frustration that often occur upon the transition to oral feeding. It supports the infants’ interactive and social behaviors as well as considers hunger satiation cycles (12). In a recent retrospective study of over 250 infants, Thomas et al. (13) showed that cue-based feeding decreased the time to full oral feeds, increased parental involvement in the feeding process, and decreased the length of hospital stay. Morag et al. (11) reported 67 infants in whom cue-based feeding facilitated parental engagement and feeding skills.

The aim of this study was to examine the short-term outcomes regarding the transition to oral feeds, growth, and length of stay, in cue-based–fed preterm infants, when compared to traditionally volume-driven–fed infants.

## Methods

### Study design

This is a retrospective cohort study comparing feeding practices over two time periods (2017–2018 and 2019–2021).

### Study population

The study population included preterm infants born before completing 34 weeks of gestation, between the years 2017 and 2021 at the NICUs of Hadassah medical centers in Jerusalem. Infants born before 2019 were routinely fed by volume-driven, structured feeding. Since 2019, preterm infants have been fed using the cue-based feeding approach. The groups were further sub-analyzed by birth weight categories, i.e., <1,500 and 1,500–2,500 g. Definitions of small, appropriate, and large for GA are based on the World Health Organization definitions (14).

### Definitions

GA was defined as the best estimate of GA determined by last menstrual period, prenatal ultrasound, and early postnatal physical examination.

Neonatal morbidities considered included respiratory distress syndrome (RDS), transitional tachypnea of the newborn (TTN), severe retinopathy of prematurity (ROP) (stage 3 and above), necrotizing enterocolitis (NEC), intraventricular hemorrhage (IVH) grades 3–4, periventricular leukomalacia (PVL), late-onset sepsis (LOS), and bronchopulmonary dysplasia (BPD) (15). RDS and TTN were defined by the presence of a consistent chest radiograph and the need for supplemental oxygen, mechanical ventilation, and surfactant treatment. BPD was defined as oxygen dependence and/or the need for ventilation support at 36 weeks of GA. ROP was diagnosed by pediatric ophthalmologists (16), and NEC was diagnosed by clinical and radiologic characteristics compatible with Bell's criteria and included NEC stages 2 and 3 (17). IVH and PVL were diagnosed by cranial ultrasonography and were graded according to the classification of Papile (18). LOS was diagnosed by two positive blood cultures beyond the first 7 days of life.

### Feeding protocols

Trophic feeding is usually initiated in our NICUs within the first 24 h of life. Breast milk is prioritized. At the time of the study, donor milk was not available in Israel, thus formula feeding was given to infants whose mothers could not provide breast milk. Thereafter, according to the infants’ ability to absorb feeds, the volume is increased by 20–30 ml/kg/day to a maximum of 140–150 ml/kg/day. In our NICU, readiness for oral feeds, based on fussing, sucking on hands and pacifiers, stable tone, and vital signs (19), is assessed by trained NICU nurses upon arrival to 33 weeks of GA. Volume-driven feeding includes a preset volume given at scheduled intervals. Our NICU shifted from volume-driven feeding to cue-based feeding in 2019. The rationale for this transition was the impression that volume-based, time-scheduled feeding may compromise the infants’ future feeding and hunger–satiety responses and increase frustration among parents and caregivers. Accordingly, our units’ protocol is applicable to every neonate and is based on the infant's clinical status and behavioral cues and does not include a weight, weight percentile for GA, or GA limit.

The cue-based feeding approach includes a minimum and maximum feeding volume, administered every 2–4 h, according to the infants’ cues of hunger. Signs of hunger include stirring, opening of the mouth, head turning, rooting, stretching, and increased movement (20). Oral feeding is ceased upon the infants’ signs of satiety, i.e., decreased sucking, turning away, etc. If the infant has not reached the minimum preset volume during 4 h, a supplementation to the minimum volume is given via nasogastric tube. Oral feeding is ceased when the feeding nurse or occupational therapist identifies signs of satiety or when the infant is feeding for over 15–20 min. The minimum and maximum volumes are designed to supply 80–160 ml/kg/day. An infant that reaches the minimum volume orally is defined as reaching full oral feeds. During the transition period, if the infant fails to complete a minimal preset volume orally, nasogastric supplementation to the preset volume is administered, as described by Morag et al. (11). Volume-driven feeds consist of a predetermined volume given every 3 h, adding up to 140–150 ml/kg/day, regardless of the infants’ sleep/awake status or hunger/satiety cues.

Trained nurses and NICU occupational therapists assess infant readiness for oral feeding. This commonly occurs after the infants have accomplished oral feeds of at least 50% of the preset volume. Z-scores of weight changes upon discharge were calculated using the 10th percentile.

### Statistical analysis

The sample size calculation was based on the expected differences between the groups (historical volume-driven feeding vs. cue-based feeding) on the days needed to achieve full oral feeding.

A sample size of 60 infants in each group was calculated for a mean difference of 2 days or more to be statistically significant (21). Data were analyzed using SPSS statistical software. To test the association between two categorical variables, the χ^2^ test as well as Fisher's exact test were used. The comparison of a quantitative variable between the two independent groups was performed using the two-sample *t*-test or the non-parametric Mann–Whitney U-test. The non-parametric test was applied for variables that were not normally distributed. All statistical tests were two-tailed, and a *p*-value ≤0.05 was considered statistically significant.

### Outcomes

The primary outcome of this study was days to full oral feeds. In volume-driven feeds, full oral feeding is defined when the infant has reached the preset volume orally and does not require nasogastric supplementations. An infant fed by cue-based feeding is considered to have reached full feeds when he reaches the minimum preset volume.

The secondary outcomes included weight and GA at full oral feeds, average weight gain per kilogram per day, weight and GA upon discharge, length of stay, breastfeeding variables (breastfeeding comprising at least 50% of feeds), nutrition type at discharge, oxygen desaturation (<90% saturation), bradycardia (heart rate <100 bpm), and apnea events (apnea lasting >5 s) during feeds. Oxygen desaturation, apnea and bradycardia episodes were adjusted per days of hospitalization.

### Ethics statement

This study was approved by the Hadassah Medical Centre ethics committee (approval number 0233-21-HMO).

## Results

### Study groups

Data were obtained from the medical files of 240 preterm infants born before completing 34 weeks of gestation. There were 120 infants in the volume-driven feeding group and 120 infants in the cue-based feeding group. Infants were further divided by birth weight: <1,500 and 1,500–2,500 g. There were 60 infants in each arm. An analysis of volume-driven feeds and cue-based feeding was performed separately for each weight group.

### Population baseline characteristics

Baseline characteristics are depicted in Table 1. There was no statistically significant difference in GA, birth weight, sex, or weight for GA between the groups. The relatively high percentage (25%) of small for gestational age preterm infants in the very low birth weight group (<1,500 g) is comparable with the national Israeli data (Israel Center for Disease Control and The Gertner Institute Women and Children's Health Research Unit).

**Table 1:** Study population baseline characteristics.

** **	** **	Volume driven *N* = 120	Cue feeding *N* = 120	*p*-value
GA (weeks), median (±SD)	<1,500 g *N* = 60	29.29 (2.9)	29.29 (2.76)	0.38
	1,500–2,500 g *N* = 60	33.5 (1.33)	33.57 (1.17)	0.848
BW (g), median (±SD)	<1,500 g	1,270 (254.4)	1,145 (262.3)	0.129
	1,500–2,500 g	1,895 (232.2)	1,940 (266.5)	0.073
*n* (%)	<1,500 g			
AGA		42 (70%)	45 (75%)	
SGA		16 (26.7%)	15 (25%)	0.52
LGA		2 (3.3%)	0 (0%)	
*n* (%)	1,500–2,500 g			
AGA		54 (90%)	55 (91.7%)	
SGA		3 (5%)	3 (5%)	1.0
LGA		3 (5%)	2 (3.3%)	
Male gender, *n* (%)	<1,500 g	26 (43.3%)	34 (56.7%)	0.144
	1,500–2,500 g	32 (53.3%)	32 (53.3%)	1.0

AGA, appropriate for gestational age; BW, birth weight; GA, gestational age; LGA, large for gestational age; SGA, small for gestational age.

Table 2 describes the frequency of neonatal morbidities between groups. Ventilation days, RDS, TTN, BPD, severe ROP, high grade IVH, PVL, NEC, and sepsis were compared. There were statistically significantly more invasively ventilated infants in the group weighing 1,500–2,500 g fed by cue-based feeding compared with infants fed volume-driven feeds. This difference was not found in very low birth weight infants. Other morbidities were comparable between the groups.

**Table 2:** Neonatal morbidities and length of stay according to feeding method.

** **	** **	Volume driven *N* = 120	Cue feeding *N* = 120	*p*-value
Invasive ventilation (days), mean ± SD	<1,500 g *N* = 60	16 ± 22.5	19.7 ± 21.9	0.131
	1,500–2,500 g *N* = 60	1.08 ± 3.9	1.53 ± 2.56	0.025
Non-invasive ventilation (days), mean ± SD	<1,500 g	3.05 ± 6.28	5.37 ± 9.15	0.181
	1,500–2,500 g	0.2 ± 0.77	0.65 ± 1.81	0.216
RDS, *n* (%)	<1,500 g	28 (46.7%)	34 (56.7%)	0.273
	1,500–2,500 g	7 (11.7%)	11 (18.3%)	0.306
TTN, *n* (%)	<1,500 g	1 (1.7%)	7 (11.7%)	0.06
	1,500–2,500 g	5 (8.3%)	11 (18.3%)	0.107
BPD	<1,500 g	15 (25%)	24 (40%)	0.079
	1,500–2,500 g	1 (1.7%)	1 (1.7%)	1.00
Severe ROP (stage 3), *n* (%)	<1,500 g	2 (3.3%)	0 (0%)	0.496
	1,500–2,500 g	2 (3.3%)	0 (0%)	0.496
Severe IVH, *n* (%)	<1,500 g	5 (8.3%)	7 (11.7%)	0.824
	1,500–2,500 g	0 (0%)	0 (0%)	1.00
NEC stage 2 and more, *n* (%)	<1,500 g	3 (5%)	3 (5%)	1.00
	1,500–2,500 g	0 (0%)	0 (0%)	1.00
PVL, *n* (%)	<1,500 g	3 (5%)	4 (6.7%)	1.00
	1,500–2,500 g	1 (1.7%)	1 (1.7%)	1.00
Sepsis, *n* (%)	<1,500 g	4 (6.6%)	6 (10%)	0.166
	1,500–2,500 g	1 (1.7%)	0 (0%)	1.00
Length of stay (days), median (SD)	<1,500 g	61.9 ± 31.5	65.3 ± 31.7	0.534
	1,500–2,500 g	222.5 ± 11.1	21.5 ± 12.9	0.149

BPD, bronchopulmonary dysplasia; IVH, intraventricular hemorrhage; NEC, necrotizing enterocolitis; PVL, periventricular leukomalacia; RDS, respiratory distress disease; ROP, retinopathy of prematurity; severe IVH, grade 3–4; TTN, transient tachypnea of the newborn.

### Feeding and weight outcomes

**Figure 1:**
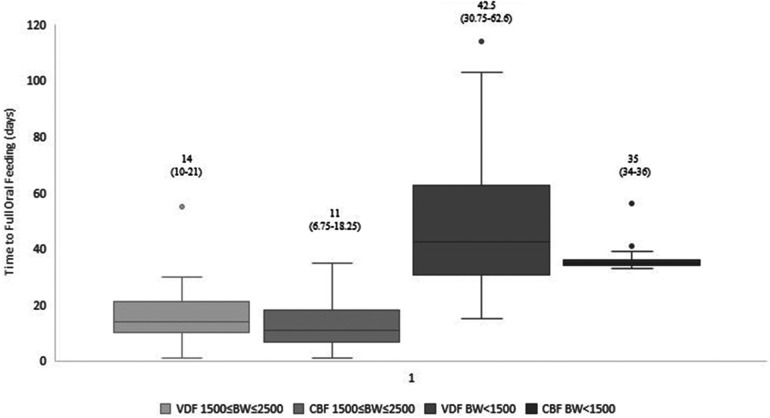
Time to full oral feeding: cue-based vs. volume-driven feeding. Data are presented as median (p25-p75). *p *=* *0.045 in the 1,500 g* *≤* *BW* *≤* *2,500 g groups. *p *=* *0.003 in the BW < 1,500 g groups. Statistical analysis was based on the Mann-Whitney test. VDF, volume-driven feeds; CBF, cue-based feeding; BW, birth weight.

**Figure 2:**
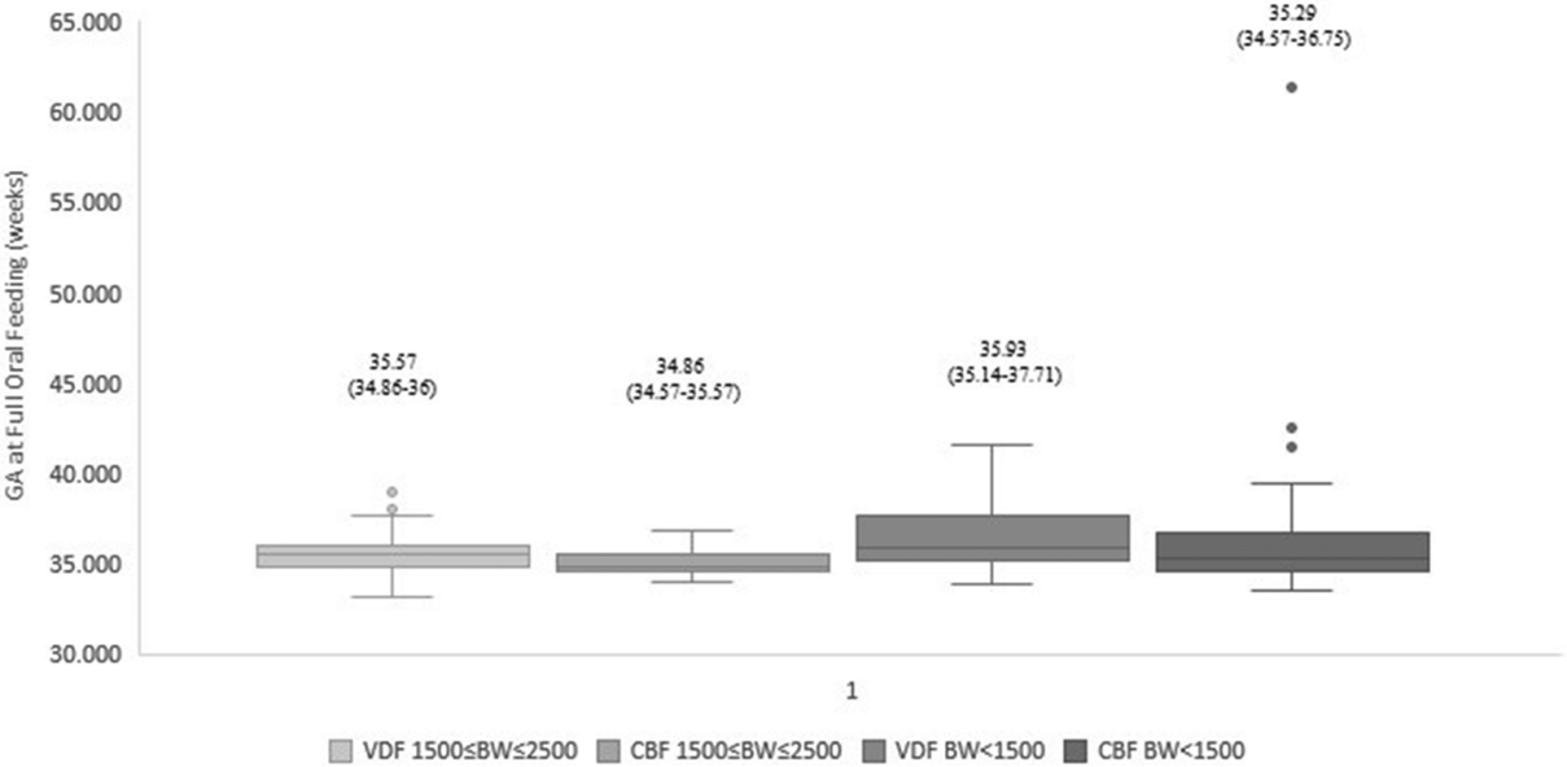
Gestational age at full oral feeds: cue-based vs. volume-driven feeding. Data are presented as median (p25-p75). *p *=* *0.004 in the 1,500 g* *≤* *BW* *≤* *2,500 g groups. *p *=* *0.012 in the BW < 1,500 g groups. Statistical analysis was based on the Mann-Whitney test. BW, birth weight; GA, gestational age; VDF, volume-driven feeds; CBF, cue-based feeding.

Our data demonstrated that infants fed by cue-based feeding reached full oral feeds earlier, both regarding days to full oral feeds and GA (Table 3 and Figures 1, 2). This finding was similar in both weight groups. Our data showed the length of stay was comparable between volume-driven and cue-fed infants. Infants born lighter than 1,500 g experienced less apnea, oxygen desaturation, and bradycardia events when fed by cue-based feeding rather than volume-driven feeds. The percentage of breastfed infants was comparable between groups. Table 4 depicts weight gain outcomes. We found that although infants with a birth weight of 1,500–2,500 g fed by cue-based feeding had a lower rate of weight gain and a more significant decrease in weight z-score from birth to discharge, absolute discharge weights did not differ. This observation was not found among the lighter infants, in whom all weight parameters were comparable.

**Table 3:** Feeding outcomes and respiratory stability according to feeding method.

** **	** **	Volume driven *N* = 120	Cue feeding *N* = 120	*p*-value
Time to full oral feeds (days), median (p25–p75)^a^	<1,500 gr *N* = 60	42.5 (30.7–62.6)	35 (34–36)	0.003
	1,500–2,500 g *N* = 60	14 (10–21)	11 (6.7–18.25)	0.045
GA at full oral feeds (weeks), median (p25–p75)	<1,500 g	35.9 (35.1–37.7)	35.3 (34.5–36.7)	0.012
** **	1,500–2,500 g	35.5 (34.8–36)	34.8 (34.5–35.5)	0.004
Breastfeeding at discharge, *n* (%)	<1,500 g	34 (56.7%)	36 (60%)	0.711
	1,500–2,500 g	34 (56.7%)	42 (70%)	0.13
Oxygen desaturation, Apnea and bradycardia events during oral feeds^b^, median (p25–p75)	<1,500 g	0.034 (0–0.097)	0.015 (0–0.053)	0.021
	1,500–2,500 g	0 (0–0.043)	0 (0–0)	0.35

^a^ p, percentile.

^b^ Adjusted for hospital stay days.

**Table 4:** Weight gain outcomes according to feeding method.

** **	** **	Volume driven *N* = 120	Cue feeding *N* = 120	*p*-value
Weight gain rate (g/kg/day)	<1,500 g *N* = 60	19.2 (15.8 to 22.1)	18.3 (16.1 to 21.4)	0.522
	1,500–2,500 g *N* = 60	8.4 (4.8 to 11.5)	5.1 (−0.6 to 9.1)	0.001
Weight at discharge (g), m (min–max)	<1,500 g	2,405 (2,105 to 2,882)	2,235 (2,097 to 2,616)	0.209
	1,500–2,500 g	2,157 (2,047 to 2,400)	2,190 (2,010 to 2,303)	0.437
Δ weight Z-score admission to discharge	<1,500 g	−0.97 (−1.46 to −0.55)	−1.21 (−1.73 to −0.73)	0.062
	1,500–2,500 g	−0.72 (−1.15 to −0.53)	−0.98 (−1.33 to −0.71)	0.004

## Discussion

In this study, we compared short-term outcomes, including time to oral feeding, weight gain, and duration of hospitalization, between cue-based and volume-driven fed preterm infants. We found that cue-based fed infants reached full oral feeds faster and at an earlier GA. Weight gain, change in weight z-score at discharge, and absolute weight upon discharge did not differ between infants with a birth weight <1,500 g. Among those whose birth weight was 1,500–2,500 g and who were cue-based fed, absolute weight at discharge was comparable; however, a slower rate of weight gain and a greater decrease in z-score from birth were demonstrated. Among this heavier group of infants, we found a higher rate of ventilation in the cue-fed infants. This finding demonstrates an advantage for cue-based feeding, even in infants with greater respiratory morbidity. Preterm infants commonly experience difficulties in transitioning from nasogastric to oral feeds. During this transition, infants may experience choking episodes, desaturation, and respiratory deterioration that may result in repeated stressful experiences to the infant and its parents. This, in turn, may lead to prolonged hospital stay, slower weight gain, and oral aversion (22). Cue-based feeding requires identifying the infant's cues of hunger and satiety, and may be performed by trained care providers, including nursing staff and occupational therapists, and parents (20). Previous studies demonstrated that parental involvement in the care and feeding of their infant increased maternal self-efficacy, the practice of neurosensory stimulation, and improves feeding and breastfeeding outcomes (6, 11, 13). In our study, we found that cue-based fed infants reached full oral feeds faster and at an earlier GA. This finding is supported by many other randomized and retrospective studies (11, 13, 23–25). Although the rate of weight gain was slower for infants with birth weights of 1,500–2,500 g fed by cue feeding in our study, the weight at discharge did not differ significantly, and the hospital stay was comparable to infants fed by volume-driven feeds. In infants with a birth weight <1,500 g, the sickest and smallest among the NICU's population, weight parameters were comparable between the groups. This may highlight the special, important advantage of cue-based feeding for this high-risk subgroup of preterm infants. Some previous studies found that preterm infants fed by cue feeding achieved improved weight gain (20, 25), while others found slower weight gain, albeit earlier full oral feeds and discharge. This inconsistency may be explained by the variation in feeding volumes, time intervals, weight and GA between centers practicing cue-based feeding (23, 26).

### Our data demonstrated comparable breastfeeding rates between groups

Previous studies, however, have shown an increase in breastfeeding rates in infants fed by cue feeding (27). This may be due to the relatively low rates of breastfeeding in our population. A recent national survey of Israeli women who gave birth to full-term infants during 2019–2020 found that 15.3% exclusively breastfed and 60% partially breastfed their infants (26). Recently, local interventions have been implemented to encourage and support mothers of preterm infants to start and continue breast milk supply in parallel with the availability of donor human milk as a bridge, until own-mothers milk supply is established. Our study demonstrated comparable breastfeeding rates between the groups, as opposed to a previous study conducted in Israel where lower breastfeeding rates were demonstrated in infants fed according to the cue-based approach (11). This result is encouraging and may reflect the change in the approach to breastfeeding in the NICU. Importantly, our data demonstrated that infants with birth weights <1,500 g experienced less oxygen desaturation, apnea, and bradycardia during cue feeding. As respiratory instability during feeds is a crucial factor in delaying discharge of preterm infants, this may have a tremendous effect on parental stress and health costs (13, 24). Increased respiratory stability during cue feeding has also been demonstrated in previous studies (11, 20, 25). As the ability to fully feed orally is a part of the discharge criteria from the NICU (28), the inability to achieve safe and efficient oral feeds often delays discharge, increasing parental anxiety and healthcare costs (13, 29–31). In previous studies, infants fed by cue feeding were found to be discharged earlier when compared to volume-driven fed preterm infants (11, 32). Our data did not demonstrate shorter hospital stays, although infants did reach full oral feeds faster in the cue-based feeding group. This implies other issues preventing discharge, such as respiratory instability or low weight. Moreover, feeding difficulties among preterm infants may continue into childhood and negatively affect their long-term outcomes, including language skills (33). As we only examined short-term outcomes, we cannot present data on this subject.

Our study has several limitations, first and foremost derived from its retrospective nature. The data were limited to medical charts, and some information, such as parental involvement in feeding, was not documented and therefore not analyzed. The strength of our study relies on the relatively large number of infants with comparable baseline characteristics and morbidities, and the sub-analysis by weight group. It has allowed us to highlight the advantages for the smallest, sickest infants, with the most prolonged hospital stay.

In conclusion, we found that cue feeding has several advantages over volume-driven feeds. These include the achievement of full oral feeds in less time and at an earlier GA, as well as less adverse effects during feeds. Staff and parental education toward a better understanding of preterm infants’ cues of hunger and satiety may decrease infant and parental stress during their NICU stay and need to be further studied.

## Data Availability

The raw data supporting the conclusions of this article will be made available by the authors, without undue reservation.
